# Associations Between Diet, Metabolic Profile, and Cognitive Function in Men with Schizophrenia and Healthy Controls: Evidence from a Comparative Study

**DOI:** 10.3390/nu18101492

**Published:** 2026-05-08

**Authors:** Krzysztof Krysta, Beata Trędzbor, Ewa Martyniak, Aleksandra Cieślik, Agnieszka Koźmin-Burzyńska, Katarzyna Piekarska-Bugiel, Rafał Bieś, Katarzyna Skałacka, Karolina Drzyzga, Marek Krzystanek

**Affiliations:** 1 Department and Clinic of Psychiatric Rehabilitation, Faculty of Medical Sciences, Medical University of Silesia, Ziołowa 45/47, 40-635 Katowice, Poland; beataziarko@poczta.onet.pl (B.T.); evamartyniak@gmail.com (E.M.); drzyzgakarolina@gmail.com (K.D.); 2 Department of Neurological and Psychiatric Nursing, Department of Neurology, Faculty of Health Sciences in Katowice, Medical University of Silesia, Ziołowa 45/47, 40-635 Katowice, Poland; acieslik@sum.edu.pl; 3 Department of Psychiatry, Department of Neurology, Faculty of Health Sciences in Katowice, Medical University of Silesia in Katowice, 40-635 Katowice, Poland; kozminburzynska@gmail.com; 4 Department of Psychiatric Rehabilitation, Leszek Giec Upper-Silesian Medical Centre, Medical University of Silesia, Ziołowa 45/47, 40-635 Katowice, Poland; kpiekarskabugiel@gmail.com; 5 Doctoral School, Department and Clinic of Psychiatric Rehabilitation, Faculty of Medical Sciences, Medical University of Silesia, Ziołowa 45/47, 40-635 Katowice, Poland; s82700@365.sum.edu.pl; 6 Institute of Psychology, University of Opole, Plac Staszica 1, 45-052 Opole, Poland; katarzyna.skalacka@uni.opole.pl; 7 Department of Clinical and Community Psychiatry and Psychology, Medical Faculty, WSB University, Cieplaka 1C, 41-300 Dąbrowa Górnicza, Poland; krzystanekmarek@gmail.com

**Keywords:** schizophrenia, diet quality, cognitive function, metabolic profile

## Abstract

**Introduction**: Growing evidence indicates that diet quality significantly influences metabolic parameters and cognitive functioning. In healthy individuals, higher consumption of minimally processed foods and products rich in omega-3 fatty acids is associated with a more favorable lipid profile and better cognitive performance. Patients with schizophrenia present an increased risk of metabolic disturbances and reduced cognitive functioning. This suggests that this group may be particularly sensitive to nutritional factors. However, relatively few studies have simultaneously examined the relationships between diet, metabolism, and cognitive profile in patients with schizophrenia and healthy individuals. **Aim**: The aim of the study was to compare the relationships between the frequency of consumption of selected food categories and metabolic parameters (glycemia, lipid profile, and insulin resistance), as well as cognitive functions (Stroop Test, Trail Making Test, and verbal fluency), in patients with schizophrenia and healthy men. **Methods**: The study included 21 patients with schizophrenia and 20 healthy men. All participants completed a questionnaire assessing the frequency of food consumption. Blood samples were collected to determine glucose, insulin, HDL, LDL, and triglyceride levels, and the HOMA-IR index was calculated. Cognitive functioning was assessed using the Stroop Test (RCNb, NCWd) and the Trail Making Test (TMT-A and TMT-B), which measure psychomotor speed and visuospatial working memory, respectively, and the verbal fluency test (semantic and phonological). Correlation analyses were performed separately in both groups. Due to the small sample size, all correlations are treated as exploratory and are analyzed with correction for multiple comparisons. **Results**: Exploratory analyses identified several patterns of associations between the frequency of consumption of selected food categories, metabolic parameters, and cognitive performance in both healthy men and patients with schizophrenia. The observed patterns differed between groups, suggesting that clinical status and treatment-related factors may modify diet–metabolism–cognition relationships. These findings highlight potential pathways linking dietary habits with metabolic and cognitive outcomes and provide a basis for further hypothesis-driven research. **Conclusions**: Diet quality may be related to metabolic status and cognitive functioning. However, the pattern of these associations differs between patients with schizophrenia and healthy individuals. The findings suggest that diet may play a role in metabolic health and cognitive functioning, particularly in clinical populations.

## 1. Introduction

Diet is one of the key environmental factors influencing both metabolic health and brain functioning. A well-balanced dietary pattern may support proper regulation of glycemia and lipid profile while also promoting neuroplasticity, neurotransmission, and protection against oxidative stress [1]. In healthy adult populations, more frequent consumption of minimally processed foods, particularly vegetables, fruits, and fish that are rich sources of omega-3 polyunsaturated fatty acids, is associated with better metabolic status and more favorable cognitive functioning [2]. In terms of potential mechanisms, omega-3 fatty acids and other bioactive dietary components are thought to influence neuronal membrane integrity, regulation of inflammatory processes, and metabolic signaling in the brain. These processes may contribute to improved synaptic plasticity and greater efficiency of cognitive functions [3].

Individuals diagnosed with schizophrenia exhibit a significantly increased risk of metabolic disturbances. These often include dyslipidemia and insulin resistance, which may be present even in the early stages of this disorder. In this population, metabolic syndrome and type 2 diabetes are also diagnosed more frequently. These associations are multifactorial and are strongly influenced by antipsychotic pharmacotherapy, which is well known to contribute to weight gain, insulin resistance, dyslipidemia, and an increased risk of metabolic syndrome and type 2 diabetes. A substantial body of evidence indicates that most second-generation antipsychotics are associated with adverse metabolic effects. These side effects significantly contribute to the elevated cardiometabolic risk observed in patients with schizophrenia. Therefore, pharmacological treatment represents one of the key contributors to metabolic disturbances in this population [4]. Behavioral factors, including lower diet quality, reduced physical activity, and higher rates of smoking, further increase the metabolic burden.

Patients with schizophrenia also show significant cognitive deficits affecting multiple domains. Impairments are particularly pronounced in executive functions, attention, and working memory, which influence the ability to plan and inhibit responses. These deficits are not limited to the disease process itself but also affect everyday functioning. As a result, they impair performance in social, occupational, and self-care domains, contributing to a reduced quality of life [5]. Because both metabolic and neurobiological factors may interact with these deficits, diet may represent an additional modifiable factor that can influence risk or provide protection. Therefore, the analysis of dietary patterns appears particularly important in this population. It may help to identify potential nutritional interventions that could simultaneously improve metabolic health and support cognitive functioning [6].

Individuals with schizophrenia tend to follow less favorable dietary patterns compared with the healthy population. Dietary analyses often show that their diets contain relatively higher amounts of saturated fats and components derived from highly processed foods, while the intake of fiber, fruits, and vegetables remains low [7]. Several factors that promote unhealthy lifestyle behaviors are more frequently observed in individuals with schizophrenia. These include altered reward mechanisms, reduced motivation to prepare meals independently, difficulties in organizing daily activities, as well as economic or social limitations [8]. Previous research has primarily focused on overall diet quality, using indices such as the Diet Quality Index or the Mediterranean Diet Score, or on selected nutritional interventions, particularly omega-3 supplementation. These studies suggest a potential beneficial role of specific nutrients in synaptic plasticity, memory, and executive processes. However, they rarely include a detailed analysis of the frequency of consumption of specific food groups in the daily diet [9]. Moreover, the literature lacks comparative studies that simultaneously examine dietary patterns, metabolic parameters, and executive functions. Such an approach is important for understanding the complex interactions between diet, metabolism, and neurocognition in individuals with schizophrenia. This gap highlights the need for multidimensional studies that integrate these domains in a comprehensive manner.

The aim of the present study was to assess the relationships between dietary patterns, metabolic profile, and executive functions. The analysis included two groups: individuals diagnosed with schizophrenia and healthy adults. The first objective was to examine how the frequency of consumption of selected food categories such as fish and other sources of omega-3 fatty acids, fruits and vegetables, and highly processed foods is associated with metabolic parameters. These parameters included, among others, insulin resistance and lipid profile. The second objective was to determine whether the frequency of consumption of these foods is related to performance in the Stroop Test, which assesses executive functions. The study also examined whether these associations differ between the clinical group and the control group. The findings may provide a basis for developing dietary recommendations that consider both metabolic health and cognitive performance in the general population as well as among individuals with schizophrenia.

## 2. Materials and Methods

The study was designed as a comparative cross-sectional project aimed at analyzing neuropsychological, psychopathological, metabolic, anthropometric, and lifestyle-related differences between men diagnosed with schizophrenia and healthy men in the control group. This design allowed for a multidimensional assessment of patient functioning and enabled the identification of relationships between diet, lifestyle factors, pharmacotherapy, and cognitive and metabolic markers.

A total of 41 men participated in the study, including 21 patients with schizophrenia (S group) and 20 healthy men matched for age and sex (HC group). Participants were between 18 and 60 years of age. Patients were recruited from the Day Unit of Psychiatric Rehabilitation at the Department of Psychiatric Rehabilitation of the Leszek Giec Upper Silesian Medical Centre in Katowice. All patients were receiving stable antipsychotic treatment; however, detailed pharmacological data were not included in the analysis. Participants were recruited consecutively from the clinical setting and local community; no randomization or blinding was applied. Individuals in the HC group were recruited from the local community. Participants were eligible if they met all inclusion criteria. For the S group, these criteria included a clinical diagnosis of schizophrenia according to ICD-10 and age between 18 and 60 years. Exclusion criteria were the same for both groups and included neurological disorders, alcohol or other psychoactive substance dependence, and other conditions that could impair cognitive functioning or influence metabolic parameters. Written informed consent was obtained from all participants.

### 2.1. Neuropsychological Assessment

All participants underwent a neuropsychological assessment using the following tests:

1. Trail Making Test (TMT), part A and part B [10], which measure psychomotor speed (part A) and visuospatial working memory (part B), respectively.
2. Stroop Color Word Interference Test, parts RCNb and NCWd [11], which assesses reading speed (RCNb) and verbal working memory (NCWd).
3. Verbal Fluency Test, including semantic and phonological categories [12], which assesses semantic and phonemic fluency.

For the purpose of the analysis, the Stroop interference index was calculated as the difference between the completion time of the interference task (NCWd) and the baseline task (RCNb). A higher value of this index indicates a greater interference effect and therefore weaker cognitive control.

### 2.2. Psychopathological Assessment

The severity of psychopathological symptoms in the S group was assessed using the Positive and Negative Syndrome Scale (PANSS) [13].

### 2.3. Anthropometric Measurements

Height and body weight were measured using a medical scale (seca GmbH & Co. KG, Hamburg, Germany). Body mass index (BMI) was calculated as body weight in kilograms divided by height in meters squared. Waist circumference was measured using an anthropometric tape [14].

### 2.4. Biochemical Markers

Blood samples were collected from participants after an overnight fast. The following biochemical markers were assessed: fasting glucose, total cholesterol, triglycerides, HDL cholesterol, LDL cholesterol, and fasting insulin [15]. The HOMA IR index (Homeostatic Model Assessment for Insulin Resistance) was also calculated [16]. Metabolic syndrome was defined according to the International Diabetes Federation (IDF) criteria.

### 2.5. Diet and Lifestyle Assessment

All participants completed the “Questionnaire for the Assessment of Dietary Beliefs and Eating Habits for People Aged 16–65, version 1.1 KomPAN” [17]. The KomPAN questionnaire is a validated tool for the Polish population, with confirmed reproducibility in previous studies. The questionnaire assessed dietary habits, nutritional knowledge, and lifestyle factors such as sleep, smoking, physical activity, and the number of hours spent using a computer.

### 2.6. Bioethics Approval

The study protocol was approved by the Bioethics Committee of the Medical University of Silesia, decision number PCN/0022/KB1/134/19; approval date: 3 December 2019.

### 2.7. Statistical Analysis

Data were analyzed using IBM SPSS Statistics version 29 (IBM Corp., Armonk, NY, USA). Descriptive analyses were conducted to determine the baseline characteristics of the analyzed variables. For variables significantly different from normal, differences were calculated using the Mann–Whitney U test. In addition, the chi square test, Cramer’s V test, and Spearman rank correlation coefficient were used to determine relationships between variables without a normal distribution. For variables meeting the assumption of normality, the Student’s *t*-test and Pearson correlation coefficient r were applied. Correlation analyses were performed separately in the schizophrenia and healthy control groups. Correction for multiple comparisons was applied within each group using the false discovery rate (FDR) method. Effect sizes were calculated using Cohen’s d and Cliff’s delta. Missing data were excluded from all analyses, and the level of statistical significance was set at 0.05.

## 3. Results

**Figure 1:**
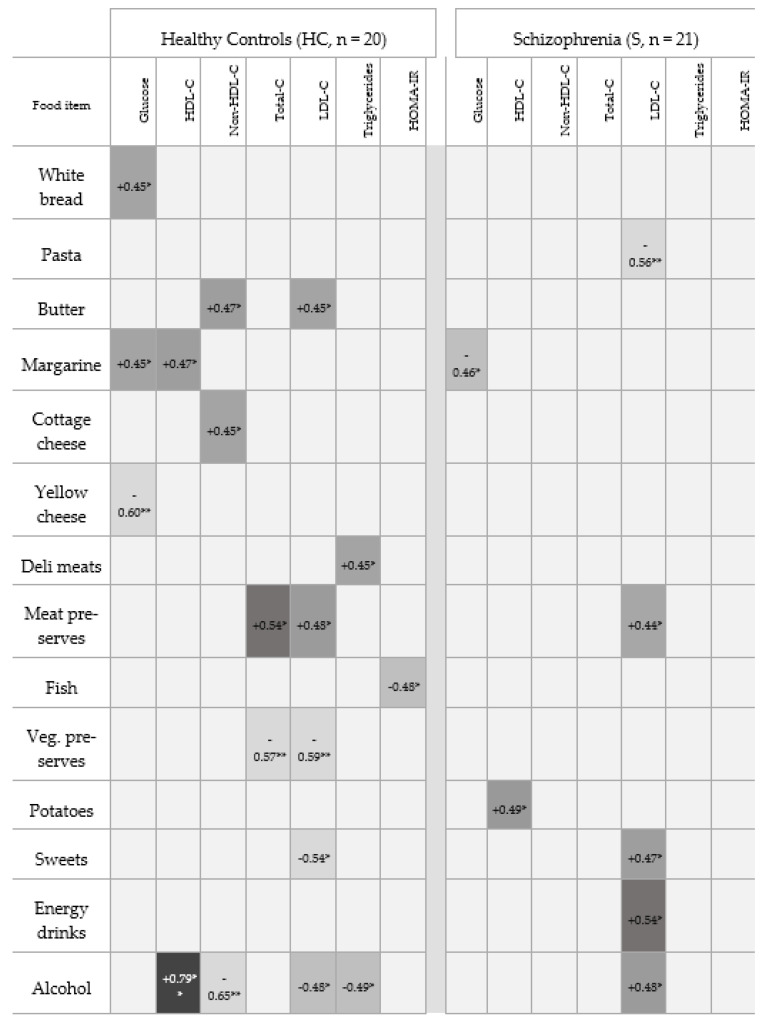
Correlations between daily food intake frequency and metabolic parameters. Legend: Dark gray cell = positive ρ|Light gray cell = negative ρ|Empty gray cell = not significant (*p* ≥ 0.05)|* *p* < 0.05 ** *p* < 0.01|Values shown as ρ (Spearman); (It should be noted that after correction for multiple comparisons using the FDR method, none of the observed correlations remained statistically significant in either group, except for the association between alcohol consumption and HDL-C in healthy participants). Abbreviations: HDL-C = HDL-cholesterol; Non-HDL-C = total cholesterol minus HDL-C; LDL-C = LDL-cholesterol; HOMA-IR = insulin resistance index (HOMA-IR). All *p*-values uncorrected for multiple comparisons.

A total of 41 men participated in the study, including 21 patients with schizophrenia (S group) and 20 healthy men (HC group). The groups differed in their metabolic and cognitive profiles (see Table 1). Correlation analysis between the frequency of consumption of selected food categories, metabolic parameters, and the results of tests assessing executive functions, processing speed, and other domains of cognitive functioning revealed different patterns of associations in the clinical and control groups (see Figure 1). It must be noted that all correlations reported below represent uncorrected exploratory findings. After correction for multiple comparisons using the false discovery rate (FDR) method, none of these associations remained statistically significant, except for the association between alcohol consumption and HDL cholesterol levels in the healthy control group.

### 3.1. Cereal Products

**Figure 2:**
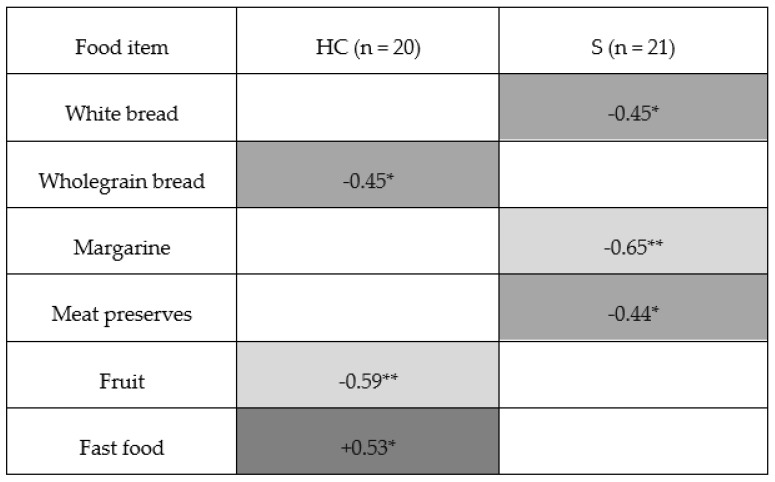
Correlations between daily food intake frequency and Stroop interference score. Legend: Dark gray = positive ρ (higher intake → worse Stroop performance)|Light gray = negative ρ (higher intake → better performance)|white = not significant (*p* ≥ 0.05)|* *p* < 0.05 ** *p* < 0.01. (It should be noted, that in both analyzed groups, none of the observed correlations remained statistically significant after correction for multiple comparisons using the FDR method.).

In the HC group, more frequent consumption of white bread was associated with higher glucose levels (rho = 0.45; *p* = 0.046—see Figure 1). In the S group, more frequent consumption of white bread was associated with a lower Stroop interference score (rho = −0.45; *p* = 0.041—see Figure 2). More frequent consumption of whole grain bread was associated with a lower Stroop interference score in the HC group (rho = −0.45; *p* = 0.046—see Figure 2), while no significant association was observed in the S group. Pasta consumption was not associated with metabolic parameters in the HC group. In the S group, a higher frequency of pasta consumption was associated with lower LDL levels (rho = −0.56; *p* = 0.008—see Figure 1).

### 3.2. Fats and Dairy Products

In the HC group, more frequent consumption of butter was associated with higher non HDL cholesterol levels (rho = 0.47; *p* = 0.036—see Figure 1) and higher LDL levels (rho = 0.45; *p* = 0.047—see Figure 1). More frequent consumption of cottage cheese was associated with higher non HDL cholesterol levels (rho = 0.45; *p* = 0.048—see Figure 1). Different patterns were observed for the consumption of oils or margarine. In the HC group, more frequent consumption was associated with higher HDL levels (rho = 0.47; *p* = 0.038—see Figure 1) and higher glucose levels (rho = 0.45; *p* = 0.047—see Figure 1). In the S group, more frequent consumption of margarine was associated with a lower Stroop interference score (rho = −0.65; *p* = 0.001—see Figure 2) and lower glucose levels (rho = −0.46; *p* = 0.038—see Figure 1). Consumption of cheese was associated with lower glucose levels only in the HC group (rho = −0.60; *p* = 0.005—see Figure 1). However, as this association did not remain statistically significant after correction for multiple comparisons, it should be interpreted as preliminary and hypothesis-generating.

### 3.3. Meat Products and Processed Foods

In the HC group, more frequent consumption of processed meat products was associated with higher triglyceride levels (rho = 0.45; *p* = 0.049—see Figure 1). Consumption of canned meat products was associated with higher total cholesterol levels (rho = 0.54; *p* = 0.014—see Figure 1) and higher LDL levels (rho = 0.48; *p* = 0.031—see Figure 1). In the S group, more frequent consumption of canned meat products was associated with higher LDL levels (rho = 0.44; *p* = 0.046—see Figure 1) and a lower Stroop interference score (rho = −0.44; *p* = 0.049—see Figure 2).

### 3.4. Fish Consumption

In the HC group, more frequent fish consumption was associated with a lower HOMA IR index (rho = −0.48; *p* = 0.032—see Figure 1). No significant associations were observed in the S group.

### 3.5. Fruits, Vegetables, and Plant Preserves

In the HC group, more frequent fruit consumption was associated with a lower Stroop interference score (rho = −0.59; *p* = 0.006—see Figure 1). No significant association was observed in the S group. In the clinical group, higher potato consumption was associated with higher HDL levels (rho = 0.49; *p* = 0.023—see Figure 1). Consumption of preserved vegetables was associated with lower total cholesterol levels (rho = −0.57; *p* = 0.009—see Figure 1) and lower LDL levels (rho = −0.59; *p* = 0.006—see Figure 1) in the HC group.

### 3.6. Highly Processed Products

In the HC group, more frequent fast food consumption was associated with a higher Stroop interference score (rho = 0.53; *p* = 0.017—see Figure 2). No significant associations were observed in the S group.

### 3.7. Sweets and Energy Drinks

More frequent consumption of sweets was associated with lower LDL levels in the control group (rho = −0.54; *p* = 0.014—see Figure 1) and higher LDL levels in the S group (rho = 0.47; *p* = 0.030—see Figure 1). Consumption of energy drinks was associated with higher LDL levels in the S group (rho = 0.54; *p* = 0.012—see Figure 1), while no significant associations were observed in the HC group.

### 3.8. Alcohol Consumption

In the HC group, more frequent alcohol consumption was associated with higher HDL levels (rho = 0.79; *p* < 0.001—see Figure 1), lower non HDL cholesterol levels (rho = −0.65; *p* = 0.002), lower triglyceride levels (rho = −0.49; *p* = 0.030—see Figure 1) and lower LDL levels (rho = −0.48; *p* = 0.033—see Figure 1). In the S group, more frequent alcohol consumption was associated with higher LDL levels (rho = 0.48; *p* = 0.034—see Figure 1). No significant correlations were found between the analyzed food categories and the results of the Trail Making Test (TMT A and TMT B). It must be noted that after correction for multiple comparisons using the false discovery rate (FDR) method, only the association between alcohol consumption and HDL cholesterol levels remained statistically significant in the healthy control group.

### 3.9. Meal Pattern

The number of meals and the regularity of meal consumption were not associated with cognitive test results or metabolic parameters in either group.

### 3.10. Metabolic Syndrome

Metabolic syndrome was diagnosed in 2 of 20 healthy men (10 percent) and in 7 of 21 patients (33 percent).

## 4. Discussion

The aim of the present study was to determine how the frequency of consumption of selected food categories is associated with metabolic parameters and cognitive functioning in two groups: men diagnosed with schizophrenia and healthy volunteers. The results indicate that certain dietary components are related both to lipid profile and glucose metabolism, as well as to selected cognitive domains. However, given the relatively small sample size and the exploratory nature of the study, these associations should be interpreted with caution. The analyses were designed to identify potential patterns rather than provide definitive evidence. In particular, the large number of correlation tests increases the risk of chance findings, and only the most robust associations may reflect stable relationships. The most consistent associations concerned the ability to inhibit automatic responses assessed by the Stroop Test. In contrast, the associations related to the Trail Making Test were less pronounced. No consistent relationships were observed for verbal fluency. Importantly, the direction of these associations differed between the clinical and control groups. This suggests that clinical status, treatment, and lifestyle factors may modify the relationships between diet, metabolism, and neurocognitive functioning. Importantly, due to the cross-sectional design of the study, all observed relationships should be interpreted as associations only, and causal inferences or assumptions about directionality cannot be made.

In contrast to Stroop interference, no significant associations were identified between dietary variables and Trail Making Test performance. This may indicate that processing speed is less sensitive to dietary influences or more strongly determined by disease-related or pharmacological factors in this population. In the control group, more frequent consumption of nutrient rich foods was associated with better psychomotor speed. In the clinical group, these relationships were weaker or inconsistent. This may indicate that this cognitive domain is more susceptible to the influence of disease related factors and pharmacotherapy. This observation is consistent with evidence indicating that information processing speed is one of the most sensitive cognitive domains in schizophrenia and may be modulated by both metabolic and neurobiological factors [18,19].

The present findings can be interpreted in the context of contemporary models of executive functions that emphasize their multidimensional nature. Increasing evidence indicates that executive functions do not represent a single homogeneous construct but include relatively distinct, although related, processes such as response inhibition, working memory updating, and cognitive flexibility. The concept of unity and diversity proposes that individual components of executive functions are correlated but remain functionally distinct, which means that cognitive performance cannot be explained solely by a single mechanism of cognitive control [20,21]. Our results support the concept of multifactorial regulation of executive functions and indicate an important role of interactions between metabolic processes and environmental factors in clinical populations. By integrating data on dietary patterns, metabolic parameters, and cognitive functioning in patients with schizophrenia and healthy individuals, this study expands the framework of metabolic psychiatry and suggests a more complex model of cognitive regulation. These findings are consistent with the growing body of evidence indicating that cognitive impairment in schizophrenia is multifactorial and remains closely related to metabolic processes [18,19]. Increasing evidence also suggests that metabolic factors and neuroinflammatory processes may play an important role in modulating executive functions in this clinical population [22,23]. The observed associations may reflect a multilevel influence of metabolic factors on cognitive functioning through mechanisms related to neuroinflammation, disturbances in insulin signaling, and modulation of the dopaminergic system [18,22,23,24].

One of the most consistent findings was the observation that in the control group more frequent fish consumption was associated with lower insulin resistance. Previous evidence indicates that dietary sources of omega-3 fatty acids, as well as fish proteins and micronutrients, may reduce insulin resistance and support better glycemic control [25]. Review studies also emphasize that regular fish consumption improves the lipid composition of cellular membranes, reduces inflammatory processes, and may modulate the gut–brain axis, which may further strengthen the protective metabolic effect. Our observations are consistent with previous evidence suggesting a potential role of fish consumption in metabolic regulation in healthy individuals. However, most of these associations did not remain significant after correction for multiple testing and should therefore be interpreted as preliminary and hypothesis.

An important relationship was also observed for highly processed products. In the control group, a higher frequency of fast food consumption was associated with poorer Stroop Test performance, which reflects weaker response inhibition and reduced efficiency of executive functions. In addition, in the group of patients with schizophrenia a significant positive correlation was observed between energy drink consumption and LDL levels. This result may indicate a potentially unfavorable influence of highly processed products rich in simple sugars and technological additives on lipid profile in the clinical population. Although this observation requires confirmation in larger studies, it is consistent with reports suggesting that high consumption of sugar sweetened beverages may contribute to metabolic disturbances. The literature indicates that diets rich in saturated fatty acids, simple sugars, and highly processed foods may impair attention, working memory, and cognitive control [26]. The relationship observed in our study is consistent with these findings and suggests that the influence of highly processed products on executive functions may occur even at moderate differences in consumption.

In the area of metabolic health, we also confirmed well documented associations between processed meat consumption and unfavorable lipid parameters. In the control group, more frequent consumption of processed meats and canned meat products was associated with higher levels of LDL cholesterol and triglycerides. Similar relationships have been described in population and clinical studies in which processed meat increased the risk of dyslipidemia and other metabolic disturbances [27]. These associations may be related to the high sodium content, preservatives, and saturated fats present in processed meats; however, they should be interpreted with caution given the exploratory nature of the study. In the clinical group, some of these products were associated with a lower Stroop interference score. The direction of this relationship is unclear and should be interpreted with caution, as it may reflect statistical artifacts, confounding, or sample size limitations rather than a robust effect. Notably, several of these associations did not remain significant after correction for multiple comparisons and should therefore be considered hypothesis-generating rather than confirmatory. It may also be related to characteristics of the diet of treated patients or the influence of pharmacotherapy on cognitive functioning. This finding may also be interpreted in the context of models of executive functions indicating that inhibitory control alone is not sufficient to explain cognitive performance, which remains the result of interactions between multiple neurocognitive and metabolic processes [20,21].

Some apparently contradictory results were also observed, including better Stroop Test performance associated with more frequent consumption of preserved foods and the absence of correlations in the group of patients with schizophrenia. These findings should be interpreted with caution and may reflect the influence of confounding factors, limited statistical power, and the exploratory nature of the study [28,29]. Additionally, the use of a frequency-based dietary questionnaire limits the precision of dietary assessment and precludes detailed mechanistic conclusions regarding specific nutrients or energy intake. The absence of significant relationships observed in the group of patients with schizophrenia may indicate disruption of typical links between diet, metabolic functioning, and cognition. This observation is consistent with reports indicating a moderating influence of chronic pharmacotherapy and neurobiological characteristics of the disorder on the physiological response to dietary factors. The possibility of reverse causality typical for observational studies also cannot be excluded [30]. A particularly stable relationship was observed between fruit consumption and Stroop Test performance in the control group. Higher frequency of fruit consumption was associated with better task performance, which suggests a beneficial effect of diets rich in polyphenols, antioxidants, and micronutrients. Although the literature does not refer directly to the Stroop Test, studies on diets rich in fruits and vegetables indicate that such products may improve memory functions, attention, and information processing speed [31]. Our data therefore provide additional support for the neuroprotective role of plant based diets.

The results related to the consumption of sweets and alcohol revealed an interesting difference between the groups. In the control group, higher alcohol consumption was associated with higher HDL levels and lower triglycerides, which is consistent with evidence regarding moderate alcohol consumption in healthy individuals [27]. Notably, after correction for multiple comparisons, the association between alcohol consumption and HDL levels was the only relationship that remained statistically significant in the healthy control group. This finding is consistent with well-established evidence indicating that moderate alcohol consumption is associated with increased HDL cholesterol levels in healthy individuals and therefore likely reflects a robust metabolic effect rather than a novel observation of the present study. Similarly, the consumption of sweets showed different associations with LDL levels in the two groups. These findings suggest that clinical factors, pharmacotherapy, or altered metabolism may modify the effects of these products on health. Such variability requires further investigation because it may reflect differences in metabolic regulation, dietary composition, or other environmental factors. Moreover, the fact that no other dietary associations remained significant after correction underscores the exploratory character of the study and suggests that many observed relationships may be context-dependent or weakened when stringent statistical control is applied.

It is also important to emphasize that the number of meals and the regularity of meal consumption were not associated in our study with any of the analyzed metabolic parameters or with Stroop Test performance. The available literature indicates that the importance of meal frequency itself may be smaller than previously assumed, while diet quality, composition, and nutrient density appear to be more important for metabolic health [32]. Our results are consistent with this perspective and suggest that the rhythm of eating alone does not play a decisive role in metabolic or executive functioning.

In summary, the results of this study indicate that selected food categories including fish, fruits, highly processed products, and processed meat may be associated with both metabolic functioning and Stroop Test performance. At the same time, differences in the direction of associations between the clinical and control groups emphasize that the influence of diet is not universal and may depend on health status, treatment, and individual dietary behaviors. These findings highlight the need for further prospective and interventional studies that could clarify whether the observed relationships are causal and which neurobiological and metabolic mechanisms may explain them. In the group of individuals with schizophrenia, several significant correlations were observed between selected elements of the diet and metabolic parameters or Stroop Test performance, but they did not form a consistent pattern. Importantly, when correction for multiple comparisons was applied, the majority of previously observed associations between diet, cognitive performance, psychopathological severity, and metabolic parameters did not remain statistically significant. This indicates that these relationships were weak and highly sensitive to statistical control. The absence of statistically robust associations may reflect a combination of limited statistical power, clinical heterogeneity, and the influence of confounding factors such as chronic antipsychotic treatment, lifestyle variability, and disrupted metabolic regulation, particularly in patients with schizophrenia. This suggests that, in patients with schizophrenia, the mechanisms linking dietary habits with metabolic and cognitive functioning may be disrupted or weakened. This observation finds partial support in the literature. In the study Dietary Behaviors and Metabolic Syndrome in Schizophrenia Patients [6], conducted exclusively in patients with schizophrenia without a healthy control group, no significant associations were found between positive dietary habits and cognitive or metabolic variables. By including a control group of healthy volunteers, our study allows comparison of mechanisms operating in the clinical and general populations, which highlights an important strength of our research design. In practice, this may indicate that this clinical group requires more specialized and targeted nutritional strategies and interventions than the general population. It should be noted that in patients with schizophrenia, the less consistent pattern of associations may be influenced by confounding factors such as chronic pharmacotherapy, smoking, or reduced physical activity, which were not formally controlled in the present analyses.

The present study has several limitations that should be considered when interpreting the results. First, the relatively small sample size may have limited the statistical power of the analyses and increased the risk of random findings, which is a well described problem in neurobiological and clinical research [28]. Second, the study included only men, which limits the generalizability of the results to the broader population. The cross-sectional design does not allow conclusions about causal relationships and does not exclude the possibility of reverse causality. An additional limitation is the use of self-report in the assessment of diet, which may be associated with recall errors and response bias. The use of a frequency-based questionnaire without quantitative dietary data limits precise estimation of nutrient intake and precludes detailed mechanistic interpretation. Another limitation is the use of a non-quantitative food frequency questionnaire, which did not allow precise estimation of the amount of consumed products or the total energy value of the diet. Memory-related errors in recalling dietary habits may also be more pronounced in individuals with schizophrenia due to the cognitive deficits observed in this population. Furthermore, the influence of pharmacotherapy was not examined in detail, although it may substantially modulate both metabolic parameters and cognitive functioning. Finally, although correction for multiple comparisons was applied in response to the exploratory nature of the analyses, some findings—particularly those with marginal *p*-values—may still reflect random variation rather than stable effects.

In conclusion, the obtained results suggest the potential importance of including targeted dietary counseling as a component of comprehensive care for patients with schizophrenia, particularly in the context of the high risk of metabolic disturbances in this population [33]. Although several significant correlations between selected dietary factors and metabolic parameters or Stroop Test performance were observed in the group of individuals with schizophrenia, they did not form a consistent pattern of relationships. Systematic monitoring of metabolic parameters and integration of psychiatric care with dietary interventions may contribute to improvements in both physical health and cognitive functioning [34]. The present observations also highlight the need for randomized interventional studies evaluating the effects of dietary modification on cognitive functioning and metabolic parameters in schizophrenia, which could provide stronger causal evidence [35].

## 5. Conclusions

1. This study provides an exploratory overview of relationships between dietary habits, metabolic parameters, and cognitive functioning in men with schizophrenia and healthy controls.
2. The observed patterns suggest that diet–metabolism–cognition relationships may differ between clinical and non-clinical populations and may be influenced by disease-related and treatment-related factors.
3. Although the identified associations require cautious interpretation, they offer important hypothesis-generating insights and underline the need for larger, longitudinal and interventional studies to clarify the role of diet in metabolic and cognitive health.

## Figures and Tables

**Table 1:** Baseline characteristics of study participants.

Variable	HC (*n* = 20)	S (*n* = 21)	*p*-Value	Effect Size
Anthropometric Characteristics [Mean ± SD; *t*-test]				
Age (years)	38.40 ± 9.70	37.90 ± 7.13	0.853	+0.06
Body weight (kg)	86.20 ± 12.29	98.38 ± 15.49	0.008 **	−0.87
Height (cm)	181.45 ± 5.49	181.67 ± 6.19	0.906	−0.04
Waist circumference (cm)	96.10 ± 8.40	105.00 ± 12.95	0.013 *	−0.81
BMI (kg/m^2^)	26.14 ± 3.40	30.01 ± 4.77	0.005 **	−0.93
Metabolic Parameters [Median (Q1–Q3); Mann–Whitney U test]				
Fasting glucose (mg/dL)	100 (92–102)	87 (81–90)	0.886	−0.876
Total cholesterol (mg/dL)	212 (164–237)	214 (168–261)	0.725	−0.936
HDL-cholesterol (mg/dL)	53 (34–67)	46 (40–48)	0.031 *	−0.607
Non-HDL-cholesterol (mg/dL)	177 (108–196)	166 (122–220)	0.814	−0.957
LDL-cholesterol (mg/dL)	108 (95–182)	127 (111–172)	0.557	−0.786
Triglycerides (mg/dL)	95 (65–152)	144 (86–199)	0.279	−0.467
Fasting insulin (μU/mL)	7.5 (5.0–15.8)	11.3 (6.8–19.1)	0.016 *	−0.562
HOMA-IR	1.71 (1.17–3.90)	2.13 (1.36–4.12)	0.022 *	−0.583
Cognitive Assessment [Mean ± SD; *t*-test]				
Stroop RCNb—time (s)	15.00 ± 3.37	20.00 ± 6.83	0.005 **	−0.92
Stroop NCWd—time (s)	27.10 ± 7.79	36.24 ± 18.60	0.049 *	−0.63
Stroop interference score (s)	−12.10 ± 7.24	−16.24 ± 16.10	0.299	+0.33
TMT-A (s)	33.15 ± 13.00	47.76 ± 20.80	0.010 *	−0.84
TMT-B (s)	85.10 ± 39.17	105.10 ± 44.33	0.135	−0.47
Semantic fluency—category 1	19.10 ± 4.81	16.05 ± 4.72	0.047 *	+0.64
Semantic fluency—category 2	14.20 ± 3.04	11.38 ± 3.67	0.011 *	+0.84
Semantic fluency—category 3	10.95 ± 2.72	10.62 ± 5.06	0.797	+0.08
Phonological fluency—letter 1	9.75 ± 3.29	10.43 ± 4.17	0.567	−0.18
Phonological fluency—letter 2	11.55 ± 4.50	12.33 ± 5.50	0.621	−0.16
Phonological fluency—letter 3	11.15 ± 3.79	10.48 ± 3.75	0.570	+0.18
Dietary Indices [Mean ± SD; *t*-test]				
Healthy diet index	4.03 ± 2.33	3.91 ± 1.49	0.843	+0.06
Unhealthy diet index	5.68 ± 1.70	6.00 ± 1.80	0.555	−0.18
Psychopathology—PANSS [Mean ± SD] (S group only)				
PANSS Total score	N/A	66.86 ± 8.31	N/A	—
PANSS Positive subscale (P)	N/A	14.29 ± 3.33	N/A	—
PANSS Negative subscale (N)	N/A	19.81 ± 4.50	N/A	—
PANSS General subscale (O)	N/A	30.71 ± 5.93	N/A	—
PANSS Supplementary (S)	N/A	3 (3–3)	N/A	—

**Notes:** *t*-test for normally distributed variables (Shapiro–Wilk *p* > 0.05 in both groups). Mann–Whitney U test for metabolic parameters. Where Levene’s test showed unequal variances, Welch’s *t*-test result is reported. * *p* < 0.05; ** *p* < 0.01. **Effect sizes:** Cohen’s d reported for *t*-test comparisons; Cliff’s delta for Mann–Whitney U test. Interpretation: |d| < 0.50 small, 0.50–0.79 medium, ≥0.80 large; |δ| < 0.147 small, 0.147–0.329 medium, 0.330–0.473 large, ≥0.474 very large. **Abbreviations:** HC = healthy controls; S = schizophrenia group; BMI = body mass index; HOMA-IR = Homeostatic Model Assessment for Insulin Resistance; RCNb = reading color-named words; NCWd = naming ink color of words; TMT = Trail Making Test; Non-HDL-C = total cholesterol minus HDL-C; PANSS = Positive and Negative Syndrome Scale; N/A = not applicable.

## Data Availability

The data presented in this study are available in the Supplementary Materials. The dataset has been fully anonymized in accordance with ethical and data protection regulations.

## Supplementary Materials

The following supporting information can be downloaded at: https://www.mdpi.com/article/10.3390/nu18101492/s1, Supplementary File S1: Anonymized_Dataset.
